# Nurse-patient relationship and its implications for retention in the PMTCT of HIV programme in Ghana: an appreciative inquiry

**DOI:** 10.1186/s12912-023-01615-z

**Published:** 2023-11-30

**Authors:** Susanna Aba Abraham, Sheila E. Clow

**Affiliations:** 1 Division of Nursing and Midwifery, Faculty of Health Sciences, University of Cape Town, Cape Town, South Africa; 2 Department of Public Health, School of Nursing and Midwifery, College of Health and Allied Sciences, University of Cape Coast, Cape Coast, Ghana

**Keywords:** PMTCT, Nurse-patient relationship, Client-Provider Interaction, Retention, Appreciative Inquiry

## Abstract

**Background:**

Relationships established between nurses and midwives, and their patients have far-reaching implications; the most significant being their impact on the health-related outcomes of patients. These relationships are especially relevant in the Prevention of Mother-to-Child Transmission (PMTCT) of Human Immunodeficiency Virus (HIV) programme as women, diagnosed with HIV navigate the emotional and psychological effects of their diagnosis while carrying pregnancies. This study aimed to explore the relationships between nurses, midwives and mothers diagnosed with HIV and its impact on retention in the PMTCT Programme.

**Methods:**

An Appreciative Inquiry approach that employed qualitative research methods was conducted among twenty-four participants made up of 12 HIV positive mothers, and eight midwives and four community health nurses engaged in the PMTCT programme. Individual generative interviews were conducted among the mothers while paired interviews were conducted among the health professionals. Thematic analysis guided by Colaizzi’s approach was conducted.

**Results:**

Three main themes emerged each with its subthemes. Under *Establishing Rapport*, two sub-themes emerged; making the connection and building trusting relationships. The second theme, *Journeying Together*, describes how the nurse-patient relationship evolved as the participant engaged in the programme; sub-themes include developing mutual goals, impactful communication, and showing commitment and building self-worth. The third theme; *Ending the professional relationship* details two sub-themes; continuity of care across the cascade, and termination of care which proved unsuccessful in some relationships due to blurring professional boundaries.

**Conclusions:**

The nurse-patient relationship in the PMTCT programme evolved as the relationship progressed along the PMTCT cascade. Strengthening of the nurse-patient relationships was underscored by building trust through the maintenance of confidentiality, setting mutual goals, shared emotional experiences and personal stories, and building clients’ self-worth. Therefore, there is a need to ensure that professional boundaries are set and maintained to reduce the occurrence of over-dependence of the clients and burnout of the nurses.

**Supplementary Information:**

The online version contains supplementary material available at 10.1186/s12912-023-01615-z.

## Introduction

Global records indicate that approximately 38.4 million people were living with HIV at the end of 2021 [1]. Most recent available data indicates that women are estimated to carry the highest burden of HIV, comprising approximately 63% of all new HIV infections recorded in sub-Saharan Africa in 2020 [2]. With most of these women in the reproductive age (WIRA), about 1.2 million women living with HIV became pregnant and gave birth during 2021, resulting in an average mother-to-child transmission (MTCT) rate of 6.2% [3].

The decline in the incidence of MTCT has been largely due to rapid scale-up of the Prevention of Mother-to-Child Transmission (PMTCT) of HIV programmes and the introduction of the Option B + approach [4] which has increased access and uptake of antiretroviral therapy (ART). However, attaining elimination of paediatric HIV seems to elude most countries as retention of women who initiate ART during pregnancy and during the postpartum period is relatively low [5].

In Ghana, PMTCT services are integrated with existing maternal, neonatal and child health services [6]. The models of care applied in the PMTCT program are client-focused and family-centered [7] which allows for incorporating the holistic and humanistic principles of health, maintenance, health promotion, client education, counseling, advocacy, and collaboration [8].

The health workers who provide PMTCT services are critical to the success of the programme. Midwives and Community Health Nurses primarily lead the provision of services in the PMTCT programme [9]. Through their engagement with their clients, therapeutic relationships are established. Pullen and Mathias [10] describe these relationships as “helping relationships” where the patient receives health-related assistance through the nurses’ knowledge and skills. The concept of nurse-patient relationship as applied in the Nursing and Midwifery professions is grounded in Peplau’s Theory of Interpersonal Relations which postulates that the nurse-patient relationship influences patients’ care experiences [11]. Thus, relationships established between nurses and midwives, and their patients have far-reaching implications; the most significant being its impact on the health-related outcome of patients [12].

These relationships are especially relevant in the PMTCT of HIV programme as women, diagnosed with HIV navigate the emotional and psychological effects of their diagnosis while carrying pregnancies [13]. These interactions between nurses and midwives, and their clients are postulated to generate into a relationship that fosters the development of trusting and therapeutic relationships. Trusting provider-patient relationships have been found to be positively associated with adherence to antiretrovirals (ARVs) and consequently retention in HIV care [14] which is critical for the prevention of vertical transmission. Morse [15] however suggested that, not all nurse-patient relationships are therapeutic in nature and those that are not, have negative repercussions such as disengagement from care and low acceptance of health provider’s advice [16]. Consequently, findings from the phase one of this study recorded the retention rate in the PMTCT programme in the study setting at six weeks postpartum as 67.4%, with the highest disengagement of 58.9% occurring antenatally [17]. In spite of the significant attrition rate, although it is uncertain, it is believed that positive experiences of nurse-patient relationships may have contributed to the mothers’ decision to remained in the programme.

This study therefore explores the care relationship between nurses, midwives and mothers diagnosed with HIV in the PMTCT Programme and how it impacts retention in the programme. It also provides insights into the dynamics of nurse-patient relationships and infers the need for tailoring nursing education and practice to consider interventions that acknowledge the unique needs of pregnant women and new mothers living with HIV to promote retention.

## Methods

### Parent study

This project employed a mixed method sequential explanatory study to explore retention in the PMTCT programme. It was organised as a two-staged study that included a retrospective cohort study and an Appreciative Inquiry (AI) that was further organised into four phases; initiate, inquire, imagine and innovate. The full study design is described in the thesis [18]. This current study is the “inquire” phase of the AI process that sought to stimulate the sharing of experiences as well as reflections on the life-giving factors [19] that promoted the nurse-patient relationships in the PMTCT programme.

### Study design and participants

The study was designed as an Appreciative Inquiry that adopted qualitative explorative descriptive research design to explore relationships that developed between the nurses, midwives and their clients in the PMTCT programme and its impact on retention. This design was appropriate as the study sought to affirm the strengths and factors that contributed to the existing nurse-patient relationship in the study setting with the intention of building on it [20] to improve retention in the PMTCT programme.

### Population and sampling

The population included women who had tested positive to HIV in the PMTCT programme during the peripartum period, and nurses and midwives whose core responsibilities included provided PMTCT services.

The mothers were eligible to participate in the study if they had remained in the PMTCT programme throughout pregnancy until the sixth postpartum week and had presented their babies for DNA PCR testing for HIV. A total of 29 mothers met the inclusion criteria. The midwives acted as gatekeepers and assisted in the recruitment of eligible mothers into the study.

On the other hand, the nurses and midwives should have provided care in the programme for at least one year at the time of data collection to be eligible for the study. Twelve midwives and six community nurses met the inclusion criteria. Recruitment was conducted by contacting them in-person to discuss the purpose of the study. Using purposive sampling, 12 mothers, eight midwives and four community health nurses were enrolled in this phase of the AI process.

### Study sites

The study was conducted in the maternity unit, comprising the antenatal, labour and postnatal units, and child welfare clinics at a Metropolitan hospital in Ghana. The facility was selected because at the time of the study, it provided the full complement of the PMTCT interventions, including counselling, testing and ART treatment within the IMNCH services. Also, being a public health facility and a secondary referral health facility, the characteristics of the clients that patronised the PMTCT services addressed the general population dynamics. The approach for treatment was option B + as per the national protocol.

### Data collection

Ordinarily, participants in AI sessions engage together starting with paired interviews [21]. However, in this study, to ensure the mothers’ confidentiality in relation to their HIV diagnosis, two approaches for data collection were employed: individual and paired generative conversations [22]. Using a semi-structured guide, face-to-face individual generative conversations were held with women infected with HIV to elicit information about their relationships with the midwives and community health nurses providing care within the PMTCT programme and to explore how their interactions with these health workers impacted their decision to remain in the programme. A total of 12 individual generative conversations were conducted based on the qualitative principle of saturation [23] at places suggested by patients including their homes and the hospital. The individual generative conversations were conducted in Fanti; a native Ghanaian language, or English at the preference of the mothers. Each participant chose a pseudonym.

Further to this, paired interviews were conducted with the nurses and midwives to elicit their experiences of establishing and maintaining relationships with their clients in the PMTCT programme. These interviews were conducted in English and transcribed verbatim. The generative conversations lasted between thirty to forty-five minutes and were audio-recorded to enable the researcher ensure an accuracy that could not be obtained from memory or field notes [24]. Field notes were also recorded to give perspective to the data during analysis.

### Trustworthiness of the study

Trustworthiness was ensured by following the constructs proposed by Lincoln and Guba [25]. An audit trail of methodological decisions was maintained throughout the study to allow for confirmability while a detailed description of purposively sampled participants was done to ensure transferability. To guarantee dependability, member checking and back translation was done to ensure meaning of data was not lost in translation, and peer review was done to validate the themes.

### Data management and analysis

The data unravelling was conducted manually and followed the approach for thematic analysis prescribed by Colaizzi [26]. Analysis was conducted inductively. The researchers began the analysis by repeatedly listening to the tapes and reading the transcripts. Transcripts in Fanti were translated into English and later back translation was conducted by the Primary Investigator (PI). As a native Fante speaker, the PI was in a position of being a translator of language, meaning and the culture that permeated through the narratives. Following this, statements that were noteworthy were identified and extracted from the dataset and imputed in a thematic analysis tracking map. Meanings were then generated from the statements. Thereafter, the formulated meanings were sorted into categories. Finally, clusters of the categories that reflected particular trends of thought were merged to form sub-themes and the themes.

## Findings

### Sociodemographic characteristics of participants

Twenty-four women participated in this phase of the AI process; 12 mothers, eight midwives and four community health.

Of the twelve who were health professionals, four were community health nurses while eight were midwives. With the exception of one community health nurse who was 56 years, all the other participants were within the reproductive age group The number of years of experience ranged between 2 and 13 years (Mean = 5.25 years; Mode = 3 years). The aggregated length of experience of the health professionals was 64 years. They provided PMTCT and MNCH services at the antenatal, postnatal and labour units along the cascade. The profile of the health professionals is presented in Table 1.

**Table 1:** Profile of health professionals

Pseudonym	Age	Status in PMTCT	Unit	Experience in PMTCT (years)
Afua	43	Midwife	Labour	5
Araba	31	Midwife	Antenatal/ Postnatal	3
Adwuba	45	Midwife	Antenatal/ Postnatal	13
Baaba	33	Midwife	Antenatal/ Postnatal	2
Ekua	28	Midwife	Labour	4
Ekuba	30	Midwife	Antenatal/ Postnatal	2
Esi	43	Midwife	Labour	8
Yaa	29	Midwife	Labour	3
Abena	34	CHN	Public Health	3
Adwoa	31	CHN	Public Health	4
Akosua	56	CHN	Public Health	5
Ama	43	CHN	Public Health	11

At the time of the interview, all the mothers were at the postnatal phase of the PMTCT cascade. Eight of the mothers had disclosed their HIV status to either their husbands or to an immediate family member. All the mothers had remained in the PMTCT programme following their HIV positive diagnosis, with the shortest length of engagement being seven months recorded by three clients. The profile of the mothers is presented in Table 2.

**Table 2:** Profile of mothers

Pseudonym	Age	HIV Disclosure status	Status in PMTCT	Phase in PMTCT cascade	Period of enrolment in PMTCT (months)	
Blessing	20	Yes	Client	Postnatal	9	
Comfort	27	Yes	Client	Postnatal	11	
Esther	33	Yes	Client	Postnatal	8	
Felicia	25	Yes	Client	Postnatal	7	
Grace	26	Yes	Client	Postnatal	10	
Irene	35	Yes	Client	Postnatal	10	
Jane	32	No	Client	Postnatal	8	
Lydia	38	Yes	Client	Postnatal	7	
Mary	34	No	Client	Postnatal	9	
Mercy	36	No	Client	Postnatal	9	
Rejoice	27	No	Client	Postnatal	8	
Vera	35	Yes	Client	Postnatal	7	

### Emergent theme and sub-themes

The findings revealed that although initial moments of the nurse-client interactions in the programme were affected by the mothers’ unexpected diagnosis of HIV in their current pregnancy, participants recounted incidents that they felt were noteworthy and enhanced their experiences in the PMTCT programme. Three main themes emerged from the analysis; *Establishing Rapport, Journeying Together and Ending the professional relationship*. Subthemes were developed under each theme and presented in Table 3.

**Table 3:** Emergent theme and sub-themes

Theme		Sub-theme
**One**	Establishing Rapport	i. Making the connection
		ii. Building a trust relationship
**Two**	Journeying Together	i. Developing mutual goals
		ii. Impactful communication
		iii. Showing commitment: Going the extra mile
		iv. Building self-worth
**Three**	Ending the professional relationship	i. Termination of the professional relationship
		ii. Continuity of care across the cascade

### Theme one: establishing Rapport

This theme describes the initial encounter between the nurses, midwives and the expectant mothers, and how the positive HIV results of the mothers’ influenced their interactions in the PMTCT programme. Two sub-themes emerged; making the connection and building trusting relationships.

#### • Making the connection

For most of the participants, their first encounters were at the antenatal booking when patients were assigned to the midwives for provision of perinatal care. In most instances, the relationships were mainly professional but the actual connection that set the stage for the engaging provider-patient relationships were made following the positive test results. It was evident from the narratives that, becoming aware of a positive HIV test at the same time impacted both the health providers and the clients and created an avenue for giving and receiving of support.

“She [midwife] was there even when they told me, I was positive. She held me as I cried and comforted me… then they [nurse and midwife] went with me to the pharmacy to begin my treatment.” Mercy, client.

For the midwives and nurses however, the offer of care although a professional mandate, was underscored by the desire to provide the needed support to see the infected women and their families through the periods of difficulty: A participant said:

“In one instance, I sat behind my desk looking at this small girl [teenager], sitting with anxiety written all over her… I felt sad but I had to tell her she was HIV positive.” Midwife Baaba.

“Although it [PMTCT service] is part of my duties as a midwife, I knew I had to put in extra effort to ensure this girl stayed… see, she was just 16 years. So, I told her, I wanted to be her friend… and she said OK.” Midwife Afua.

#### • Building trusting relationships

From the participants stories, trusting relationships were established between the nurses and midwives and their clients in the PMTCT programme. The nurses and midwives’ maintaining patients’ confidentiality was a key indicator for the establishment of the trusting relationship. Most of the patients tested the nurses and midwives’ commitment to their needs and fully committed to the relationship when the nurses and midwives showed signs of being trustworthy .

“She [pregnant woman] told me that, she had not disclosed her status [HIV] so she does not want me to inform her sister. So, I reassured her that I will not expose her. But I realized that she was still worried… I am sure she was waiting to see if her sister will get any hint [about her status] from me… Now when she [pregnant woman] calls… she tells me, I am wonderful and when I ask her why she says that I thought you would disclose my status [to pregnant woman’s sister], a positive relationship has developed between us up till now. I know that to me and the client this is exceptional.” Midwife Afua.

Another important consideration of the mothers for committing to the provider-patient relationship in the PMTCT programme was the use of positive affirmation by the health workers for the mothers’ effort of adhering to treatment and keeping all appointments

“She [community health nurse] told me how she admired my seriousness in taking my treatment to protect my child. She encouraged me and reassured me that if I continued that way, my baby would be negative. She made me feel cared for that day…” Mary, client.

Being accepting and non-judgmental was a key feature that emerged from the data as essential for the building of a trusting relationship between the midwives, nurses and their clients.

“We show love to the clients and accept [them]. So, that they feel they can establish a trusting relationship with you. This makes the women open and can discuss their challenges with us by calling you or even coming to the facility just to see you. This makes them stay because they realize that they may not receive that kind of treatment elsewhere.” Community health nurse Akosua.

### Theme two: journeying together

The relationship forged between the nurses, midwives and the pregnant women evolved as the engaged in the programme; mutual goals were developed, had impactful communication and these contributed to building the mothers’ self-worth.

#### • Developing mutual goals

From the narratives, it was evident that interactions between the midwives, nurses and the mothers centered around developing mutual goals. These goals centered mainly on maintaining the health of the mother and preventing vertical transmission.

“… She [midwife] told me that we had to plan for the child, how we can ensure that the child will not get the disease… She told me to agree to take the drugs to reduce my viral load so that the baby will not get infected… Jane, client.

“My main concern was to make sure that people did not notice that I had HIV. When I told her this, she also said it was her goal too. She then talked about the drugs and why I had to take it as she had taught me” Lydia, client.

Further to this, the narratives revealed that the health workers also took ownership of the goals and this shared effort and dream was an important reason why the nurse-patient relationship evolved.

“We also planned on how to ensure the baby will not acquire the virus. That is my job, that is my goal. So, I discuss this with the mother so that they know we are in this [PMTCT] together” Midwife Yaa.

#### • Impactful communication

For most of the participants, the opportunity to effectively explain their position on issues and actively listening to the other person in the relationship was helpful in fostering the decision of mothers to commit to the nurse-patient relationship as well as remain in programme.

“I [midwife]… get the person to understand what the condition [HIV] really is. I am able to get the person to understand… there is hope for her. So, if she is able to go by the rules especially with the medications, then she can move on. And when she accepts that, you [midwife] too it makes everything so easy for you, because she [client] knows that I am doing this for the reward I am going to get for myself and my baby. So, if she really understands what she is doing, then everything becomes so easy for you [midwife].” Midwife Araba.

“The nurse who counselled me told me what we needed to do to ensure my baby was born negative [HIV]. She was specific about my role… coming for all the antenatal visits, making sure I do all the labs [investigations] and not missing [drugs] even one day. She said even though it may be difficult, we had to do it to achieve our goal.” Vera, client.

Sharing the success stories in the PMTCT programme with newly diagnosed HIV positive women was an important catalyst for retention. One mother explained that the nurse sharing information about the success story of other mother-baby pairs in preventing vertical transmission was a helpful reminder to remain in care. She said:

“…She [midwife] even used other people as examples, even a nurse who tested positive in pregnancy and decided to take her treatment and now her child is 20 years and still negative… this raised my hopes up and made me desire the same thing.” Jane, client.

Additionally, participants inferred that focusing communication on positivity instead of the challenges was also an important feature in the nurse-patient relationship they experienced in the PMTCT programme.

“They [nurses] will not scare you. They will speak nicely to you so that you are encouraged and gain the confidence to take care of your baby.” Vera, client.

“Whenever we met, although she [client] had complaints and challenges, I always drew her attention to how far we have come in spite of all the struggles. I always made the effort to make both of us look at the cup half full, instead of half empty…” Midwife Adwuba.

Gradually, the narratives of the participants became positive and filled with hope and excitement instead of despair and this impacted mothers’ decision to remain in the programme.

The relationship built fostered discussions, encouragement and the decisions about disclosure of their status to others as patients became more confident to disclose to a member of their family.

…After a while, she [mother] agreed to tell her husband about the results… Midwife Araba.

#### • Showing commitment: going the extra mile

From the participants’ perspectives, observing the commitment of the other person in the nurse-patient relationship to the goals set was an important factor that promoted an unwavering relationship. Constantly reminding the clients of appointments and refills were cited by some clients as a reason for retention in the programme.

“I saw that she wanted to help me. She gave me reminders of my appointments and ART refills. Because of this, I couldn’t stay home, I had to also do my part and also follow all their instructions.” Rejoice, client.

For another client, the sacrifices by the staff which required going the extra mile to ensure that her concerns were addressed further fostered the nurse-patient relationship and underscored retention.

“When the Pharmacists went on two months strike nationwide… I needed a refill for my treatment and that of the baby. I had heard of the strike and knew the pharmacy was closed. About a day later, I met XXX outside the hospital, and I informed him that I needed a refill. He spoke to me nicely and reassured me that he would assist me. Later in the afternoon, he came to my shop and asked me to follow him. He opened the office and looked for my PMTCT folder and supplied the drugs. In truth, I appreciated him so much, because the folders were many, but he spent time looking for mine.” Vera, client.

#### • Building self-worth

The analysis revealed that the relationships between the midwives and CHNs, and the mothers’ stories revealed that not being labeled by the condition in the relationship contributed to clients building their self-worth. Psychological care included the sense of acceptance, reassurance, and encouragement that some mothers received while in the programme. A mother reported that:

“She [midwife Esi] treated me as a human being and not an HIV patient… The care the midwife gave me, made me feel special. She treated me like a human being.” Jane, client.

This built the mothers’ self-worth and confidence.

### Theme three: ending the professional relationship

As the mothers progressed through the peripartum period, they transitioned through the integrated Maternal and Child Health units, and PMTCT cascade. Thus, as they exited preceding units, some relationships they formed with nurses in those units were terminated as newer ones were established with health professionals in the units they found themselves. This theme describes how the mothers transitioned to form other nurse-patient relationships along the cascade while previous relationships ended.

#### • Continuity of care across the cascade

The analysis revealed that several nurses and midwives introduced their patients to others colleagues in subsequent units offering PMTCT services as their patients transitioned through the PMTCT cascade.

“I had already psyched her that I would not be the only person to take care of her… I had already told her that at a point in time, some people might come in…. So, I introduced her to those[CHN] at RCH [Reproductive and Child Health unit] so that when she comes, she would not have to go and explain so many things.” Midwife Araba.

It was also evident that although new relationships were formed as the mothers progressed through the cascade, the initial relationships were still maintained especially by the efforts of the mothers.

“Even though I am now at postnatal clinic, I still call X [midwife] at the antenatal clinic whenever I need her. She was very good to me and treated me like a sister. So, I still keep in touch… I never go to the hospital without visiting her.” Lydia, Client.

The narratives also revealed that in some units along the PMTCT cascade, where clients’ length of stay was short and interactions with health professionals were limited, staff did not link their clients to the subsequent unit.

“Yes, we need to link the labour ward with the PNC unit. I think we [labour ward staff] have to ensure continuity of care for up to some time until we are convinced that both mother and baby are doing well; that they come for their refills and the baby too is doing well…” Midwife Esi.

#### • Termination of professional relationship

The nurse-patient relationship within the PMTCT programme ended as the mothers exited the peripartum period and enrolled in adult ART care. For several of the midwives in the labour ward, termination was not discussed with client and was abrupt.

“It is like we end contact with the patient as soon as the client delivers.” Midwife Yaa.

For midwives in the antenatal clinic and the postnatal clinic however, clients were informed of the need for termination prior to the scheduled day.

“I educate them that when it gets to a point, you will leave me… so, I make them understand. I just don’t push them.” Midwife Baaba.

It was also evident that for some of the participants, termination was unsuccessful as although the clients moved on to adult ART programme for services, they still reverted to their relationships within the PMTCT programme when they faced challenges while accessing care.

“I was in the counselling room one day when Madam X [client] came to me… she had missed a treatment refill appointment by a few days, and so the pharmacist had refused to serve her unless she presented a treatment supporter. After counselling her on adherence, we went together to the pharmacist,” Community health nurse Adwoa.

Evidence from narratives revealed that some of the difficulties occurred because the relationships extended beyond the requirement of the PMTCT programme.

“She calls me at any time… she is so comfortable with me. And I see her in the market, she calls me and like [to say] Aunt nurse, here is your baby [Fante]. Anything with her baby, she tells me and even her life, her normal life, everything aside her condition, she tells me everything. I have bonded with her like a sister, like a relative.” Midwife Araba.

## Discussion

The study explored the nurse-patient relationships within the PMTCT programme and its implications for retention. The study findings revealed that the relationships in the PMTCT programme usually began as professional relationships, where on the initial encounter, the nurse or midwife offered a service to test the clients for HIV and the client chose to accept the offer. These initial professional relationships are sanctioned by policy that requires nurses and midwives to offer HIV Testing and Counselling (HTC) to every pregnant client at antenatal booking unless the client opts out [6]. Morse [15] describes this relationship as a *clinical relationship* as the encounter is superficial and courteous.

The study found that the shared emotional experiences of receiving a positive HIV result for the first time by both the nurses and midwives, and their clients underscored the beginning of a *therapeutic and empathetic relationship* [15] that is peculiar and intentional, and extended beyond mere service delivery. This finding corroborates the position of Schweitzer [27] who intimates that shared emotions enables both participants in the relationship to connect with their emotions, develop their ability for emotional empathy and to understand the co-experiencers. Peplau refers to this stage of the nurse-patient encounter as *orientation* and suggests that the nurses and midwives’ initial assessments of the patient needs and inclinations to experience strong emotions like fear and anxiety puts the nurse in the light of a person qualified to help the patient [28] and sets the stage for a trusting and helping relationship and retention in the PMTCT programme. The finding is important to nursing practice as it denotes the need for midwives and nurses to conduct holistic assessment of their patients and to be intentional about validating their emotional reactions to their diagnosis to be able to build a trusting relationship that can contribute to retention.

In this study, the most important catalyst for promoting trust in the nurse-patient relationship was the maintenance of confidentiality. Several studies have intimated fear of unpleasant consequences related to HIV disclosure such as isolation [29], intimate partner violence [30], fear of divorce and loss of economic support [31]. The requirement of confidentiality is guided by the ethical principles of respecting the autonomy of the client to disclose at a time she feel safe, preventing harm from others and ensuring it yields benefits to the clients [32]. Thus, the promise of confidentiality provided the sense of safety for the mothers and yielded a stronger commitment to the nurse-patient relationship and consequently promoted retention in the PMTCT programme. Consequently, it is important to institutionalise the confirmation and reiteration of confidentiality as a core principle of practice in each nurse-patient interaction in the PMTCT programme to promote the building of trust in the health staff and invariably positively impact retention.

The study findings also revealed that as the trusting relationships progressed, the nurses were able to counsel clients on the benefits of retentions and adherence to treatment protocols to both the mother and her child and by so doing developed mutual goals. Peplau describes this phase as the *working phase* of the relationship [11] when the patient sees the nurse as consistently helping, providing unconditional love and empathy. Wolf et al., reports that shared goals promotes social closeness with an interaction partner and influences their sense of connectedness [33]. Consequently, in this study, the trusting relationship evolved to the mutually dependent relationship, where the actions and inactions of both partners impacted the achievement of their mutually set goals. Morse [15] refers to this relationship between nurses and midwives and their clients as the *connected relationship*, where the nurses and midwives begin to see the women first as a persons before being patients.

The use of positive affirmation and sharing of personal success stories were evidently useful in strengthening the partnership in the nurse-patient relationship in this study. Unhjem et al., [34]

intimate that self-disclosure or sharing personal and work-related stories have beneficial effects on the therapeutic relationship between nurses and midwives, and their clients. In this instance, sharing personal stories revealed their personal vulnerabilities and struggles to the clients and reshaped the perception of the nurses as all-knowing. This contributed to rescaling the power dynamics in the relationships [35] while promoting patients’ self-efficacy. Consequently, Peplau posits that, power shifts from the nurses, tilting more towards the clients during the working phase of the nurse patient relationship [11, 28].

As the nurse-patient relationships progressed, the need to terminate the relationship occurred and required the clients established new relationships in subsequent units along the PMTCT cascade. It was evident that, although most clients were informed about the impending termination of engagement, its implementation were unsuccessful in several instances. In this study, these failed termination attempts occurred because for several of the relationships, the professional boundaries were not clearly defined. Griffith alludes to the fact that a key consideration for setting appropriate boundaries for a nurse-patient relationship is that all engagements are focused on the care needs of the patients [36]. Consequently, Morse alluded to the nurse-patient relationships that extends beyond professional boundaries as *over-involved* [15]. Although, this type of relationship may positively impact retention in care, it could result in overdependence on the nurses and midwives and consequently lead to burnout. This finding highlights the need for applying nursing care approaches that affords the health professionals the time and opportunity to engage and commit to journey with the clients within the boundaries of the profession to ensure the wellbeing of the mother and child, enhance the care experience, promote a positive image of the profession as well as enhance retention in the programme.

It was also evident that, ensuring continuity of care along the various units of the PMTCT cascade and empowering the clients to initiate new relationships as their needs changed promoted retention throughout the cascade.

### Strengths and limitations of the study

The strength of the study includes the use of AI instead of a problem-focused approach. This afforded the researchers the opportunity to focus on the strengths, celebrate achievements and envisage possibilities instead of focusing on the challenges existing in nurse-patient relationships in the PMTCT programme. However, the possibility of missing out on relevant information on the challenges in pursing the nurse-patient relationship in the PMTCT programme exists. Also, social desirability bias cannot be excluded as the participants shared some form of relationship overtime and the mothers still accessed care in the health facility and thus, they may have given responses that they felt may reflect positively on the nurses and midwives instead of their experiences. To reduce this, questions were rephrased and confidentiality was assured throughout the interviews. It is recommended that further studies are conducted in Adult ART clinics to explore the relationships building following exit from the PMTCT programme.

## Conclusion

The nurse-patient relationship in the PMTCT programme evolved as the relationship progressed along the PMTCT cascade. Strengthening of the nurse-patient relationships was underscored by building trust through maintenance of confidentiality, setting mutuals goals, shared emotional experiences and personal stories, and building clients self-worth. These positively impacted commitment to the nurse-patient relationship and also impacted retention in the programme. There is however the need to ensure that professional boundaries are set from the onset of the relationship thereby reducing the occurrence of overdependence of the clients and burnout of the nurses.

### Electronic supplementary material

Below is the link to the electronic supplementary material.

Supplementary Material 1

## Data Availability

The data and materials are available from the corresponding author upon reasonable request.

## Abbreviations

AI: Appreciative Inquiry
ART: Antiretroviral Therapy
ARV: Antiretrovirals
HIV: Human Immunodeficiency Virus
HTC: HIV testing and Counselling
IMNCH: Integrated Maternal Neonatal and Child Health
MNCH: Maternal Neonatal and Child Health
PMTCT: Prevention of Mother-to Child-Transmission
WIRA: Women in the Reproductive Age
MTCT: Mother-to-Child Transmission
