# Supplementary material for: Nurse-patient relationship and its implications for retention in the PMTCT of HIV programme in Ghana: an appreciative inquiry
Source: BMC Nurs. 2023 Nov 30;22:450. doi: 10.1186/s12912-023-01615-z (PMC10688082; doi:10.1186/s12912-023-01615-z)
Supplement: Supplementary file 1 — Supplementary Material 1 [file 12912_2023_1615_MOESM1_ESM.docx]

**Interview guide for mothers:**

Date & time of interview: Interviewer:

***Introduction***

Thank you for taking time out of your busy schedule to take part in this interview. My name is ____. Before we start, I would like to explain the focus of this interview. You were contacted because in spite of the many challenges people face in the PMTCT programme, you have kept all your appointments. I am sure that, having stayed in the PMTCT for more than six months, you have had some wonderful and trying experiences as well as established some relationships with a nurse or midwife that has help with you decision to remain in the programme. I would like to gain insight into how this relationship was established and how you worked together to make your PMTCT journey successful so that we can find ways to enhance the experiences of others enrolled in the programme to promote retention. Please remember that your feedback is confidential.

1. Looking back since your diagnosis and enrollment in the PMTCT programme, think about an experience you have had with a nurse or midwife – a time when you felt cared for and had a sense of satisfaction. Tell me a story of that experience.

*Probe*

- 1. What made that experience exceptional?

1. If I were to ask you about your choice of a “best” nurse within the PMTCT programme, who will you choose and why?
2. Can you reflect and share how you met this nurse?
3. What prompted a relationship between the two of you?

*Probe*

- 1. Can you describe the characteristics/attributes that makes this relationship special from any other nurse/midwives you have received care from other clinics?
  2. What have you contributed to make sure this relationship lasts?

1. Would you say that your relationship has extended beyond the confines of the PMTCT programme? Why do you say that?

*Probe*

- 1. Do you share social calls and engagements?
  2. Do you discuss issues outside of PMTCT care?

1. How has this relationship impacted your decision to remain in the PMTCT programme?

*Probe*

- 1. Health related decision-making.
  2. PMTCT care
  3. Continuity of care

1. Will you recall your nurse discussing ending the care relationship with you?
2. *Probe*
   1. How did you feel?
   2. What steps did the nurse/midwife take to ensure you still remained in care?
3. If you had the opportunity to talk to other nurses about establishing relationships with their clients in the PMTCT programme, what will be your best advice?

**Interview guide for Nurses and Midwives**

Date of interview:

***Introduction:***

The Appreciative inquiry process involves paired interviews and small group activities. You will also be expected to share your thought with the whole group. At the end of the sessions, I will collect all the documents you created as individuals and groups because the information constitutes an important part of the data for the study. Please do not write your name on any documents (Write the interviewee’s name as written on the name tag).

1. *Working in pairs, take turns to interview each other. Participant A interviews Participant B and vice versa.*
2. *Use the interview schedule as your script and ask the questions as they are written on the interview schedule.*
3. *Let the interviewee tell his or her story and PLEASE do not interrupt.*
4. *Take notes of high points and quotable quotes.*
5. *Check with the interviewee if you captured the highlights of the story that mattered most.*

Initiate phase

1. Reflect for a moment and remember an exceptional experience you had working in the PMTCT programme - a time when you felt you were really contributing to the lives of the women (and the babies) that you have served, a time when you felt you were really making a difference. Tell me a story about that experience.
2. What made that experience exceptional?

*Probe:*

1. Who was the patient involved and what prompted a relationship between the two of you?

*Probe*

- 1. Can you describe the characteristics/attributes that makes this relationship special from any other nurse/midwives you have received care from other clinics?
  2. What are the key factors that made this experience exceptional?

1. Can you reflect and share how you met this patient?
2. What prompted a relationship between the two of you?

*Probe*

- 1. Can you describe the characteristics/attributes that makes this relationship special from any other clinics?
  2. What have you contributed to make sure this relationship lasts?

1. Would you say that your relationship has extended beyond the confines of the PMTCT programme? Why do you say that?

*Probe*

- 1. Do you share social calls and engagements?
  2. Do you discuss issues outside of PMTCT care?

1. How has this relationship impacted your decision to continue working in the PMTCT programme?
2. Will you recall discussing ending the care relationship with the patient?
3. *Probe*
   1. How did you feel?
   2. What steps did the nurse/midwife take to ensure you still remained in care?
4. If you have three wishes that would ensure that more of these exceptional experiences / relationships would be possible more of the time, what would they be?

***Thank you for participating***
